# A Change of Scenery: Does Exposure to Images of Nature Affect Delay Discounting and Food Desirability?

**DOI:** 10.3389/fpsyg.2021.782056

**Published:** 2021-12-06

**Authors:** Katie Clarke, Suzanne Higgs, Clare E. Holley, Andrew Jones, Lucile Marty, Charlotte A. Hardman

**Affiliations:** ^1^Department of Psychology, University of Liverpool, Liverpool, United Kingdom; ^2^School of Psychology, University of Birmingham, Birmingham, United Kingdom; ^3^School of Sport, Exercise and Health Sciences, Loughborough University, Loughborough, United Kingdom; ^4^Centre des Sciences du Goût et de l’Alimentation, AgroSup Dijon, CNRS, INRAE, Université Bourgogne Franche-Comté, Dijon, France

**Keywords:** nature exposure, urban, delay discounting, food desirability, dietary restraint, mood

## Abstract

Previous research suggests that exposure to nature may reduce delay discounting (the tendency to discount larger future gains in favor of smaller immediate rewards) and thereby facilitate healthier dietary intake. This pre-registered study examined the impact of online exposure to images of natural scenes on delay discounting and food preferences. It was predicted that exposure to images of natural scenes (vs. images of urban scenes) would be associated with: (i) lower delay discounting; (ii) higher desirability for fruits and vegetables (and lower desirability for more energy-dense foods); and (iii) delay discounting would mediate the effect of nature-image exposure on food desirability. Adult participants (*N* = 109) were recruited to an online between-subjects experiment in which they viewed a timed sequence of six images either showing natural landscape scenes or urban scenes. They then completed measures of mood, delay discounting (using a five-trial hypothetical monetary discounting task) and rated their momentary desire to eat four fruits and vegetables (F&V), and four energy-dense foods. There was no statistically significant effect of experimental condition (natural vs. urban image exposure) on delay discounting or food desirability. Bayes factors supported the null hypothesis for discounting (BF_01_ = 4.89), and energy-dense food desirability (BF_01_ = 7.21), but provided no strong evidence for either hypothesis for F&V desirability (BF_01_ = 0.78). These findings indicate that brief online exposure to images of nature does not affect momentary impulsivity or energy-dense food preference, whereas for preference for less-energy dense foods, the evidence was inconclusive.

## Introduction

The physical and psychological health benefits of spending time in nature are becoming increasingly apparent. These include improvements to mood, mental health, cognitive function, and physical activity, and decreased risk of cardiovascular disease (Atchley et al., 2012; White et al., 2013; Zelenski et al., 2015; Jimenez et al., 2021). However, increasing global urbanization can reduce people’s exposure and access to green spaces thereby limiting opportunities for health benefits (Cox et al., 2018). Obesity is a major global public health issue (Wolfenden et al., 2019) and there is emerging evidence that nature exposure can increase healthy dietary behaviors (Spendrup et al., 2016; Sobko et al., 2020). A nature-based intervention study by Sobko et al. (2020) increased children’s connection to nature and found positive effects on their eating behaviors (e.g., greater consumption of vegetables) and caregiver feeding styles. Furthermore, Spendrup et al. (2016) found that exposure to recorded nature sounds (birdsong) increased willingness to buy organic foods in men but not women. However, the mechanisms explaining a potential positive effect of nature exposure on food-related behaviors are unclear.

According to Life History theory, humans respond adaptively to cues within their environment that signal both opportunity or threat (Del Giudice et al., 2016). Urban environments are thought to signal threat due to unpredictability and social competition for resources (van der Wal et al., 2013), which leads individuals to value the present more than the future, resulting in impulsive decision-making such as delay discounting (the tendency for individuals to devalue larger distant benefits in favor of smaller immediate gains) (Griskevicius et al., 2012). In contrast, natural environments are thought to signal stability thereby allowing individuals to put greater value on the future and reduce delay discounting (Griskevicius et al., 2012). Consistent with this idea, previous studies have shown that virtual (*via* photographs) and real exposure to nature reduces monetary delay discounting compared to exposure to urban environments (van der Wal et al., 2013; Berry et al., 2014).

Delay discounting is also associated with consumption of energy-dense foods and obesity (Barlow et al., 2016), potentially because the delayed benefits of healthy eating are discounted in favor of immediate reward from energy-dense foods. Relatedly, consuming a healthy diet is associated with a greater focus on future consequences (e.g., health), and a reduced focus on immediate satisfaction (Dassen et al., 2015). Given the aforementioned theoretical relationship between nature exposure and lower delay discounting (due to natural environments signaling stability and promoting future-thinking), lowering delay discounting may be a mechanism for how nature exposure can improve diet. A recent study (Kao et al., 2019) supports this premise; exposure to images of natural scenes (relative to urban images) in a laboratory context reduced participants’ consumption of sugar in a drink, and this effect was mediated by lower delay discounting. However, the extent to which this effect may generalize to other participant populations, contexts, and a broader range of foods is unknown.

The current study aimed to determine the effect of exposure to images of natural scenes, presented in an online context, on delay discounting and food preferences in participants from the United Kingdom. It was hypothesized that nature images (relative to urban images) would be associated with: (i) lower monetary delay discounting; (ii) higher desirability for fruit and vegetables (F&V) and lower desirability for more energy-dense foods; and (iii) delay discounting would mediate the effect of nature-image exposure on F&V desirability. Kao et al. (2019) only included individuals with weight loss intentions. Therefore, as a secondary research aim, we explored whether the effect of nature-image exposure on healthy food desirability is moderated by individual differences in dietary restraint.

## Materials and Methods

### Participants

Participants were recruited using opportunity sampling (e.g., university email lists and social media) to take part in this online experimental study. First year psychology students also participated in return for course credits. Inclusion criteria were being aged 18 years or over, a United Kingdom resident and fluent in English. Ethical approval was granted by the University Research Ethics Committee. As a cover story, participants were told that the study was about measuring memory and choices. The study was powered to detect a medium effect size (*d* = 0.5) based on Kao et al. (2019) using GPower 3.1, which indicated that 128 participants were required in a between-subjects design with 80% power and alpha level of 0.05. The protocol and analysis strategy were pre-registered on Open Science Framework^1^.

### Experimental Stimuli

Images were sourced from free online image databases such as https://unsplash.com. To select the stimuli, a separate sample of participants (*N* = 20; 14 female) rated 40 images of landscapes (20 images of natural scenes and 20 images of urban scenes) on the following attributes; naturalness (5-point Likert scale with anchors 1 = extremely urban to 5 = extremely natural), pleasantness (1 = extremely unpleasant to 5 = extremely pleasant), and arousal (1 = extremely relaxing to 5 = extremely exciting).

On the basis of these ratings, 12 images (six natural scenes and six urban scenes) were subsequently selected for inclusion in the main study using the criteria that natural images had mean ratings for naturalness above 3.5, and urban images had mean ratings below 2.5. Descriptive characteristics of the images selected for inclusion in the main study can be found in Table 1 (the full set of included images can be found in Supplementary Material). The six selected images of natural scenes were, on average, rated significantly higher for naturalness than the six urban images [*t*(5) = 11.96, *p* = <0. 001]. The natural images were also rated significantly higher for pleasantness than the urban images [*t*(5) = 3.47, *p* = 0.018]. The urban images were rated as significantly higher for arousal (excitement) than the natural images [*t*(5) = −2.86, *p* = 0.036].

**TABLE 1:** Descriptive statistics of images selected for each experimental condition.

	**Natural images (*N* = 6)**	**Urban images (*N* = 6)**
Naturalness (1–5)	4.14 (0.29)	1.86 (0.21)*
Pleasantness (1–5)	3.73 (0.15)	2.98 (0.57)*
Arousal (1–5)	2.41 (0.35)	2.80 (0.18)*

*Values are means with SD in parentheses.*

*Significantly different from natural images, *p* < 0.05.

### Measures

#### Delay Discounting

In an adjusting delay task (Koffarnus and Bickel, 2014), participants completed five hypothetical choice trials between a smaller amount of money (£5) now or a larger amount of money (£10) at variable delays starting with 3 weeks. Depending on the choice made in the first trial, the delay to the larger amount either adjusted up (if delayed amount was chosen) or down (if immediate amount was chosen). The outcomes are the Effective Delay 50% (ED_50_; the delay in days that effectively discounts the value of the delayed reward by 50%) and the discount rate (*k*), which is calculated as the inverse of the ED_50_. A higher discount rate (*k*) indicates less willingness to wait for the delayed higher reward and greater preference for the immediate lesser reward. Following the methodology of the authors who developed and validated the 5-choice task (Koffarnus and Bickel, 2014), we calculated the *k*-parameter and used this as the outcome variable for delay discounting.

#### Food Desirability

Four images of F&V (strawberries, raspberries, cucumber, and carrots) and four images of energy-dense foods (chocolate-covered donut, chocolate buttons, sweets, and crisps) were presented in a randomized order. Images were sourced from the food-pics image database (Blechert et al., 2019). Participants rated how much they wanted to eat that food right now on a 100 mm Visual Analog Scale (VAS), with “not at all” and “very much” as the anchors.

#### Dietary Restraint

The 10-item restraint subscale from the Dutch Eating Behavior Questionnaire (DEBQ) was used (Van Strien et al., 1986). Cronbach’s alpha (α) for current study = 0.93.

#### Mood

Using the Positive and Negative Affect Scale (PANAS) (Watson et al., 1988), participants rated their current mood in relation to 10 items measuring positive affect (e.g., excited and inspired), and 10 items measuring negative affect (e.g., upset and afraid). For the current study α = 0.62 for both the positive and negative subscales.

### Procedure

Participants clicked on a weblink to enter the study, which was hosted by Qualtrics, and firstly provided informed consent. They were then randomized to either the natural-image condition or the urban-image condition. They viewed a sequence of six images either showing natural scenes or urban scenes. Each image was shown individually on the computer screen for 30 s and participants were instructed to study the images carefully because they would be taking part in a memory test afterward. Next, participants completed the PANAS followed by three memory questions relating to the images they had just viewed (in addition to fulfilling the cover story, the memory check provided an indicator of participants’ attention to the images). The delay discounting task and food desirability tasks were then completed in a counter-balanced order followed by the DEBQ restraint scale. Finally, participants reported their age, gender, ethnicity, highest level of education, current employment status, height, weight, any currently diagnosed or historical eating disorders, and their beliefs about the study aims.

### Data Analysis

Statistical analyses were conducted in IBM SPSS (v25). An independent-samples *t*-test examined the effect of experimental condition (natural- or urban-image exposure) on mean delay discounting rate (*k*). A multivariate analysis of variance (MANOVA) examined the effect of condition on desirability for F&V, and energy-dense foods^2^. We also conducted the following pre-registered exploratory analyses: (i) MANOVA to determine the effect of image exposure on mood (PANAS positive and negative affect scales as the dependent variables) and (ii) simple moderation analysis using the SPSS PROCESS macro to determine whether the effect of image exposure on food desirability was moderated by dietary restraint. Bayes factors demonstrating support for the null hypothesis (BF_01_) were conducted in JASP (JASP team), using default priors for the natural vs. urban contrasts on (i) discount rate, (ii) F&V desirability, and (iii) energy-dense food desirability.

## Results

### Participant Characteristics

A total of 131 participants began the study. Of these participants, 109 had complete data and were included in the analyses (*N* = 56 natural condition; *N* = 53 urban condition). Descriptive data for age, gender, and other study variables can be found in Table 2. Participants were predominantly white (92%), university students (66%), and educated to a college-level qualification or above (97%). Fourteen percent reported a historical or current diagnosis of an eating disorder. The number of memory questions answered correctly (attention check) did not differ significantly between conditions [*t* = −1.54 (107), *p* = 0.126] (see Table 2).

**TABLE 2:** Participant characteristics and descriptives for main variables stratified by image exposure condition. Values are mean with SDs in parentheses unless stated otherwise.

	**Natural condition (*N* = 56)**	**Urban condition (*N* = 53)**	**Total (*N* = 109)**
Age (years)	24.77 (10.94)	24.21 (10.96)	23.85 (5.11)
Gender (female/male/other)	45/11	44/8/1	89/19/1
BMI (kg/m^2^)	23.71 (3.55)	24.03 (6.45)	24.5 (10.90)
Delay discounting rate (k)	0.09 (0.32)	0.35 (2.33)	0.22 (1.64)
F&V desirability VAS (0–100)	49.55 (20.21)	42.78 (22.66)	46.26 (21.61)
Energy-dense food desirability VAS (0–100)	46.98 (21.12)	45.51 (24.55)	46.27 (22.76)
Positive affect (10–50)	30.46 (8.75)	27.94 (9.95)	29.24 (9.39)
Negative affect (10–50)	16.30 (7.08)	17.51 (8.08)	16.89 (7.57)
Dietary restraint (1–5)	2.88 (0.97)	2.94 (0.94)	2.88 (0.95)
Number of memory questions correctly answers (0–3)	1.46 (1.08)	1.74 (0.71)	1.60 (0.92)

*BMI, body mass index; F&V, fruit and vegetables; VAS, Visual Analog Scale.*

Correlations between the variables of interest can be found in Table 3.

**TABLE 3:** Pearson’s correlation coefficients between main variables of interest.

	**F&V desirability**	**ED food desirability**	**Restraint**	**BMI**	**Positive affect**	**Negative affect**
*k*	0.02	0.07	−0.10	−0.22*	−0.00	0.17
F&V desirability	–	0.43*	−0.04	−0.07	0.01	−0.19
ED food desirability	–	–	−0.29*	−0.11	0.06	−0.10
Restraint	–	–	–	0.19	0.02	−0.02
BMI	–	–	–	–	0.01	0.12
Positive affect	–	–	–	–	–	0.17

*p < 0.05. ED, energy-dense.

### Effect of Image Exposure on Delay Discounting and Food Desirability

**FIGURE 1:**
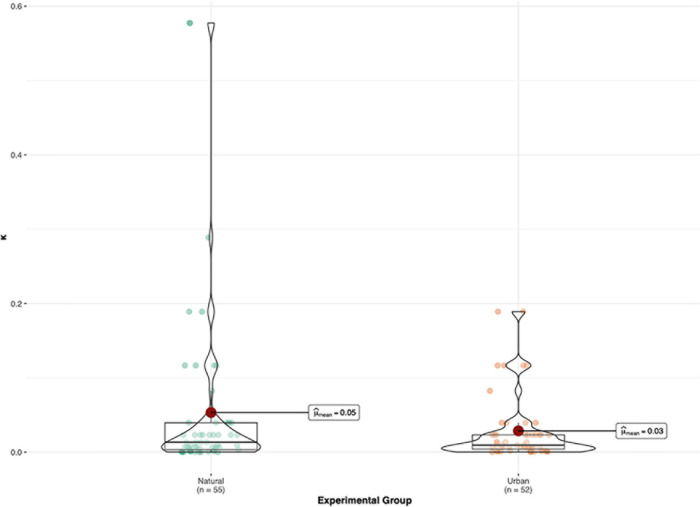
Mean differences in *k* score (delay discounting) across experimental condition (natural vs. urban). A higher *k* indicates less willingness to wait for the delayed higher reward. For *k*, 1 outlier removed from each group.

For delay discounting (*k*), the difference between the natural- and urban-image conditions was not statistically significant^3^, *t*(107) = −0.81, *p* = 0.42, *d* = −0.16. See Figure 1 for a graphical representation of the data (with outliers removed).

**FIGURE 2:**
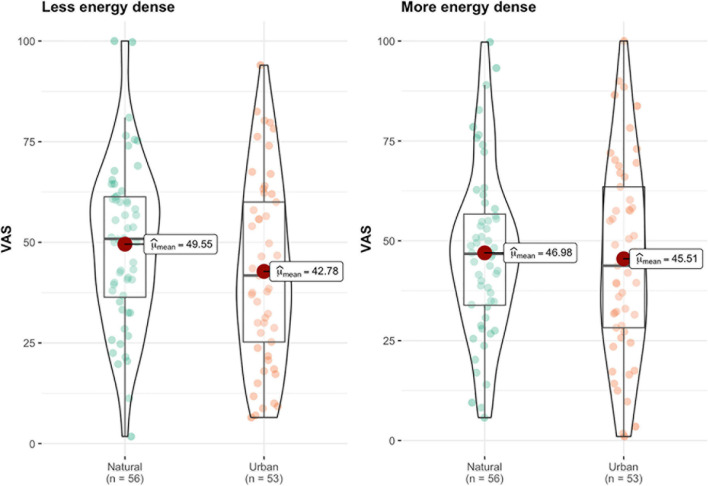
Mean differences in preference for less energy-dense (fruit and vegetables) and more energy-dense foods, measured on a 100 mm Visual Analog Scale (VAS), across experimental condition (natural vs. urban).

The MANOVA showed no overall effect of experimental condition (natural vs. urban image exposure) on food desirability (F&V or more energy-dense VAS), [*F*(2,106) = 1.43, *p* = 0.243, *$\eta_{p}^{2}=0.03$*]. See Figure 2 for a graphical representation of the food desirability data.

Bayes factors were in support of the null hypothesis for discounting (BF_01_ = 4.89), and more energy-dense food desirability (BF_01_ = 7.21), but provided no strong evidence for either hypothesis for F&V desirability (BF_01_ = 0.78).

### Exploratory Analyses

There was no significant effect of experimental condition on positive or negative mood [*F*(2,106) = 1.58, *p* = 0.210, *$\eta_{p}^{2}=0.03$*].

The moderation model, with condition as the independent variable and restraint as the moderator, predicted 4% of the variance in F&V desirability [*F*(3,105) = 1.57, *p* = 0.202]. There was no main effect of experimental condition (*b* = −23.52, SE = 13.22, *p* = 0.08) or restraint (*b* = −9.55, SE = 6.77, *p* = 0.16), and the interaction between condition and restraint was also non-significant (*b* = 5.81, SE = 4.36, *p* = 0.185) (see Supplementary Material for a visual representation of this interaction).

## Discussion

The current findings indicate that brief online exposure to images of natural scenes (relative to urban scenes) did not have a significant impact on delay discounting or food desirability ratings. This is contrary to previous research showing that nature exposure can lower delay discounting (van der Wal et al., 2013; Berry et al., 2014), and as a result promote healthier dietary choices (Kao et al., 2019).

In the current study participants were exposed to each image for 30-s, a shorter exposure time compared to some studies (e.g., 1-min exposure per image in Kao et al., 2019). Longer exposure times may be needed to achieve behavioral effects, as well as more engaging and immersive exposure such as using virtual reality, and field experiments of actual nature exposure (e.g., van der Wal et al., 2013). The current study was limited in this respect due to it being administered online to remote participants. Participant inattention and distractibility are generic issues for online studies, and our attention check revealed that, on average, participants answered fewer than two of the three memory questions correctly.

The effects of nature exposure on diet might only be apparent in certain participant groups. Kao et al. (2019) tested participants with intentions to lose weight, and this suggests that individuals who restrict their food intake in order to lose weight may be most susceptible to the effect. Contrary to this, we found little evidence that dietary restraint moderated the effect of nature exposure on desirability for more healthful foods (i.e., fruits and vegetables). However, it is important to note that these were (pre-registered) exploratory analyses and our study was not powered for formal moderation analysis.

Surprisingly, there was no effect of nature exposure on mood as measured by the PANAS. Attention Restoration Theory (Kaplan, 1995) proposes that nature may have more specific restorative benefits which replenish depleted cognitive resources. This cognitive restoration may facilitate better self-regulation, including in relation to diet. Notably, Michels et al. (2021) found that nature images were rated as higher in restorative power than urban images. Furthermore, green nature pictures specifically (relative to black–white nature) had positive effects on happiness recovery during a stress induction, suggesting that specific visual characteristics of nature exposure are important. However despite this, and consistent with our results, Michels et al. (2021) did not find an effect of nature exposure on snack food consumption.

Our study has a number of limitations. The participants were predominantly white, more highly educated young females with BMIs in the healthy range, and the extent to which these findings would generalize to other populations is unclear. The small number of males in our sample precluded the study of gender differences, however previous studies suggest there may be differences between men and women in terms of how they respond to nature-related stimuli (Spendrup et al., 2016). Due to the online delivery of the study, we were unable to use more immersive nature exposure or to measure actual food choices and intake. Our final sample size (*N* = 109) was less than that specified in our power calculation (*N* = 128) due to participant attrition during the study. However, we supplemented our main analyses with Bayes Factors which is recommended practice to improve inferences in the event of null findings (Lakens et al., 2018).

Future research is required to understand the specific format and dose of nature exposure that is needed to elicit emotional, cognitive, and behavioral outcomes that benefit health. Other potential mechanisms for how nature exposure might influence diet should also be examined; for example, people may be nudged toward healthier choices due to prior associations between nature, health and sustainable eating practices (Hagen, 2020; Krizanova et al., 2021). Connection to nature is also significantly associated with trait mindfulness (Schutte and Malouff, 2018), and therefore nature exposure could promote a more attentive approach to eating and better dietary control. Participants’ habitual exposure to nature may also moderate any effect. It will also be important to take into account participants’ current hunger state which can influence ratings of food desirability (Rogers and Hardman, 2015). Future studies should also determine whether the effect of nature exposure differs by body weight status. Given the positive effects of nature exposure on physical and mental health, the potential for benefits on diet merits further consideration.

## Data Availability Statement

The datasets presented in this study can be found in online repositories. The names of the repository/repositories and accession number(s) can be found below: https://osf.io/jsmk9/files/.

## Ethics Statement

The studies involving human participants were reviewed and approved by the University of Liverpool, Health and Life Sciences Research Ethics Committee (Psychology, Health and Society). The patients/participants provided their written informed consent to participate in this study.

## Author Contributions

KC undertook the experimental preparation and data collection. KC, AJ, and CAH performed the statistical analyses. KC and CAH wrote the first draft of the manuscript. All authors contributed to the study conception and design, commented on drafts of the manuscript, and approved the final version.

## Conflict of Interest

CAH has received research funding from the American Beverage Association, and speaker fees from International Sweeteners Association and International Food Information Council for work outside of this manuscript. The remaining authors declare that the research was conducted in the absence of any commercial or financial relationships that could be construed as a potential conflict of interest.

## Publisher’s Note

All claims expressed in this article are solely those of the authors and do not necessarily represent those of their affiliated organizations, or those of the publisher, the editors and the reviewers. Any product that may be evaluated in this article, or claim that may be made by its manufacturer, is not guaranteed or endorsed by the publisher.

## Footnotes

1
https://osf.io/6fv3g

2 The pre-registration plan outlines a mediation analysis with bootstrapping to test hypothesis iii; however, as hypotheses i and ii were not supported (see section “Results”), this analysis was redundant and therefore not conducted.

3 Removal of one substantial outlier for *k* from the urban condition did not affect this result, *t*(106) = 1.44, *p* = 0.15.
