# Supplementary material for: A Change of Scenery: Does Exposure to Images of Nature Affect Delay Discounting and Food Desirability?
Source: Front Psychol. 2021 Dec 6;12:782056. doi: 10.3389/fpsyg.2021.782056 (PMC8685291; doi:10.3389/fpsyg.2021.782056)
Supplement: Supplementary file 1 [file Data_Sheet_1.docx]

Supplementary Material

## Supplementary Figures

## Supplementary Figure 1. Images of natural scenes included in the main study.


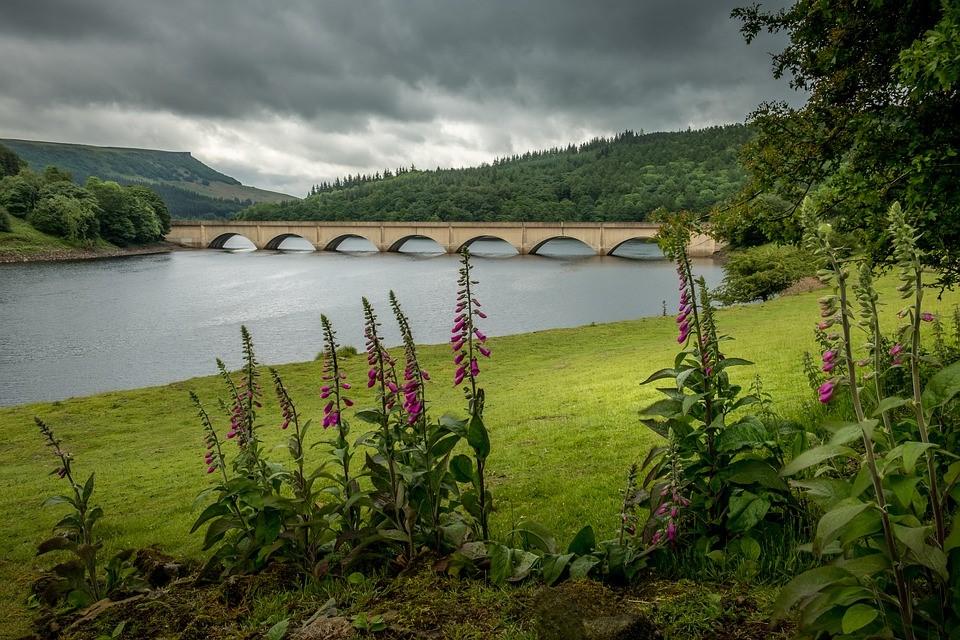


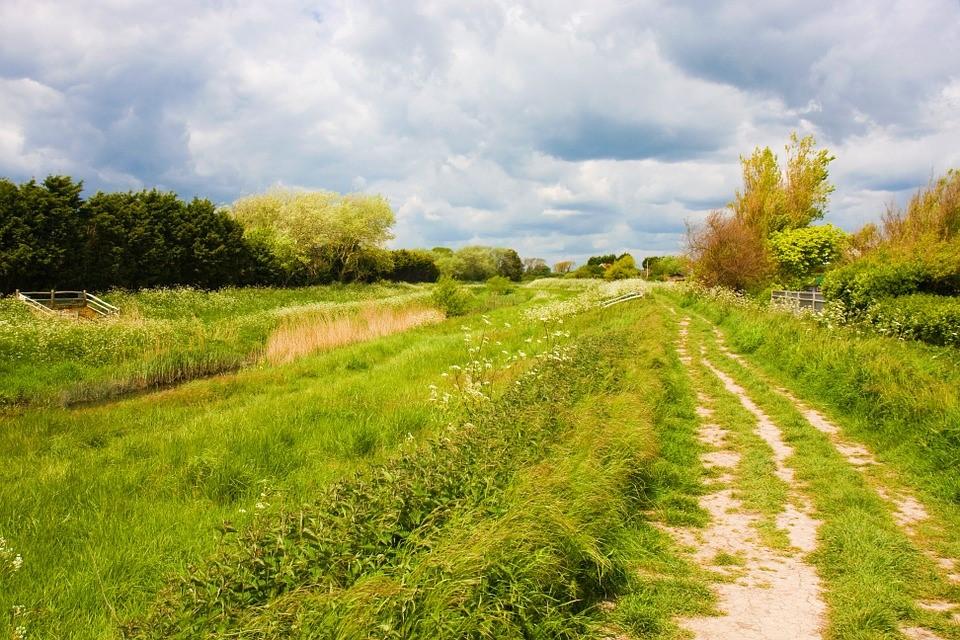


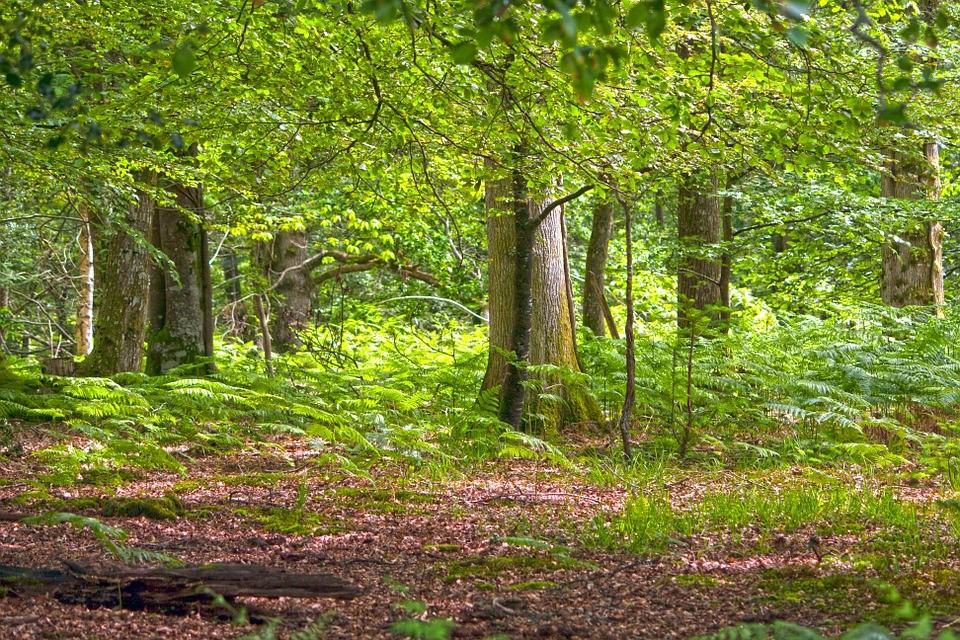


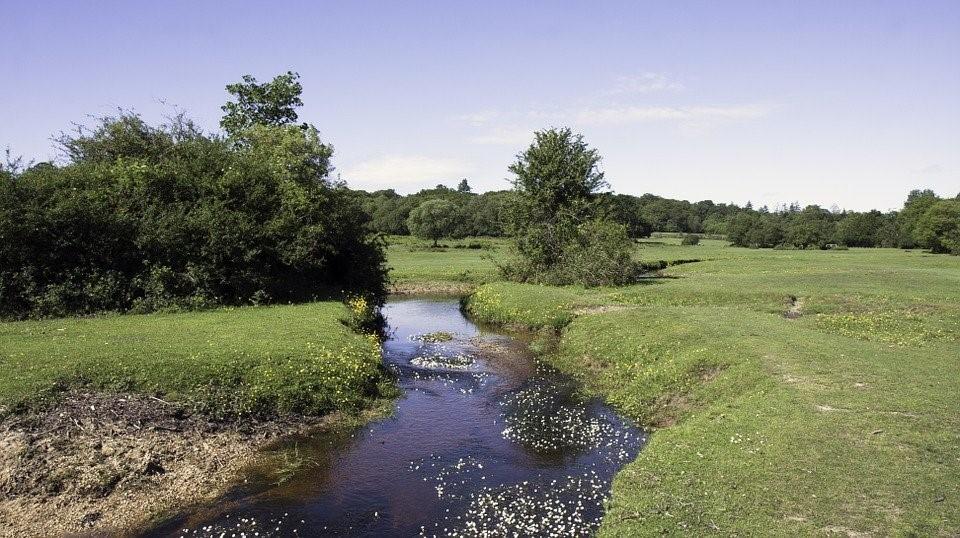


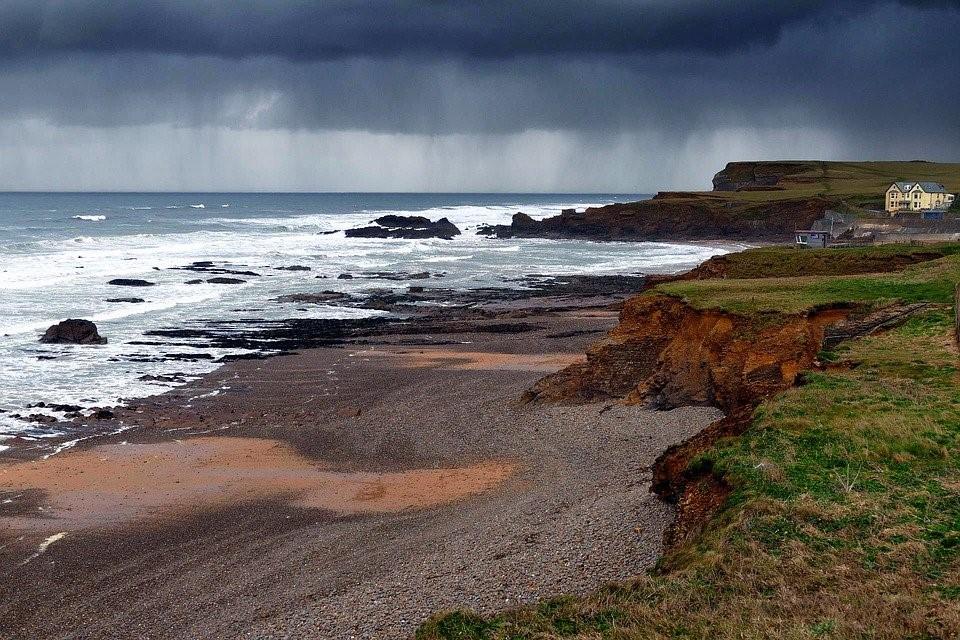


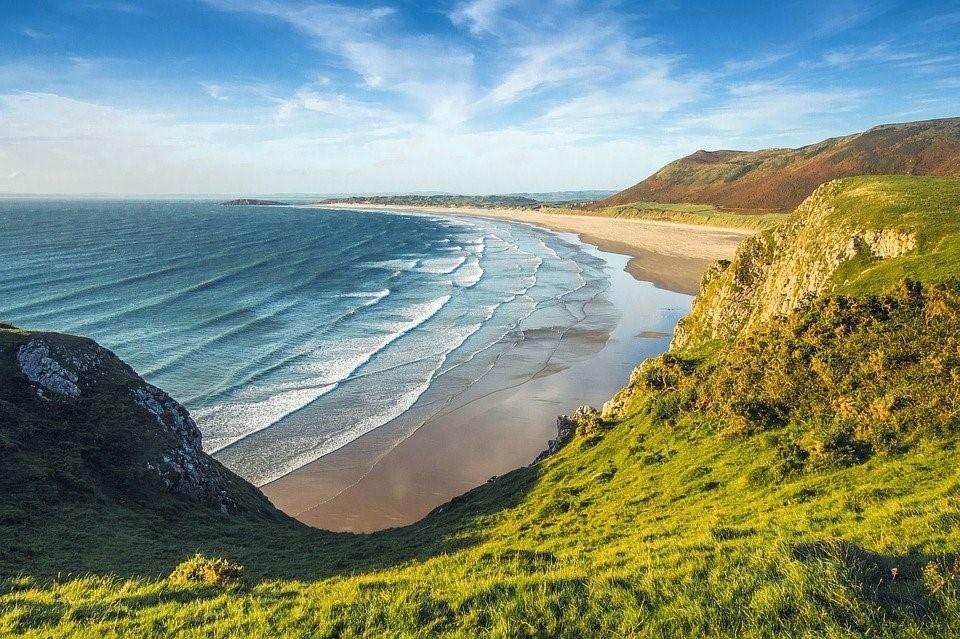


## Supplementary Figure 2. Images of urban scenes included in the main study.


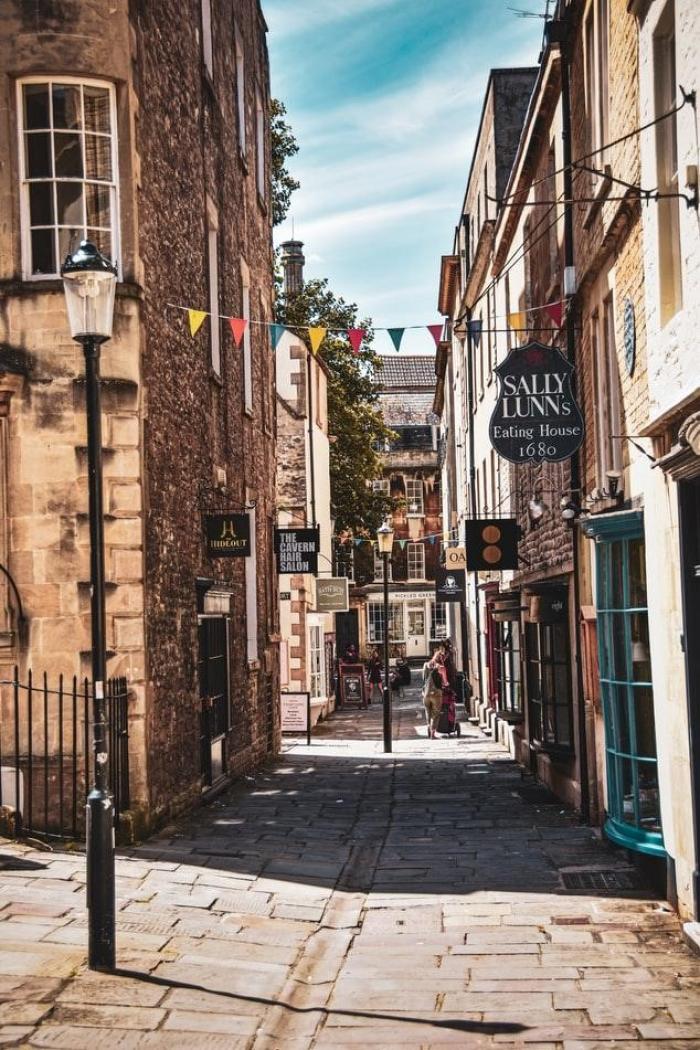


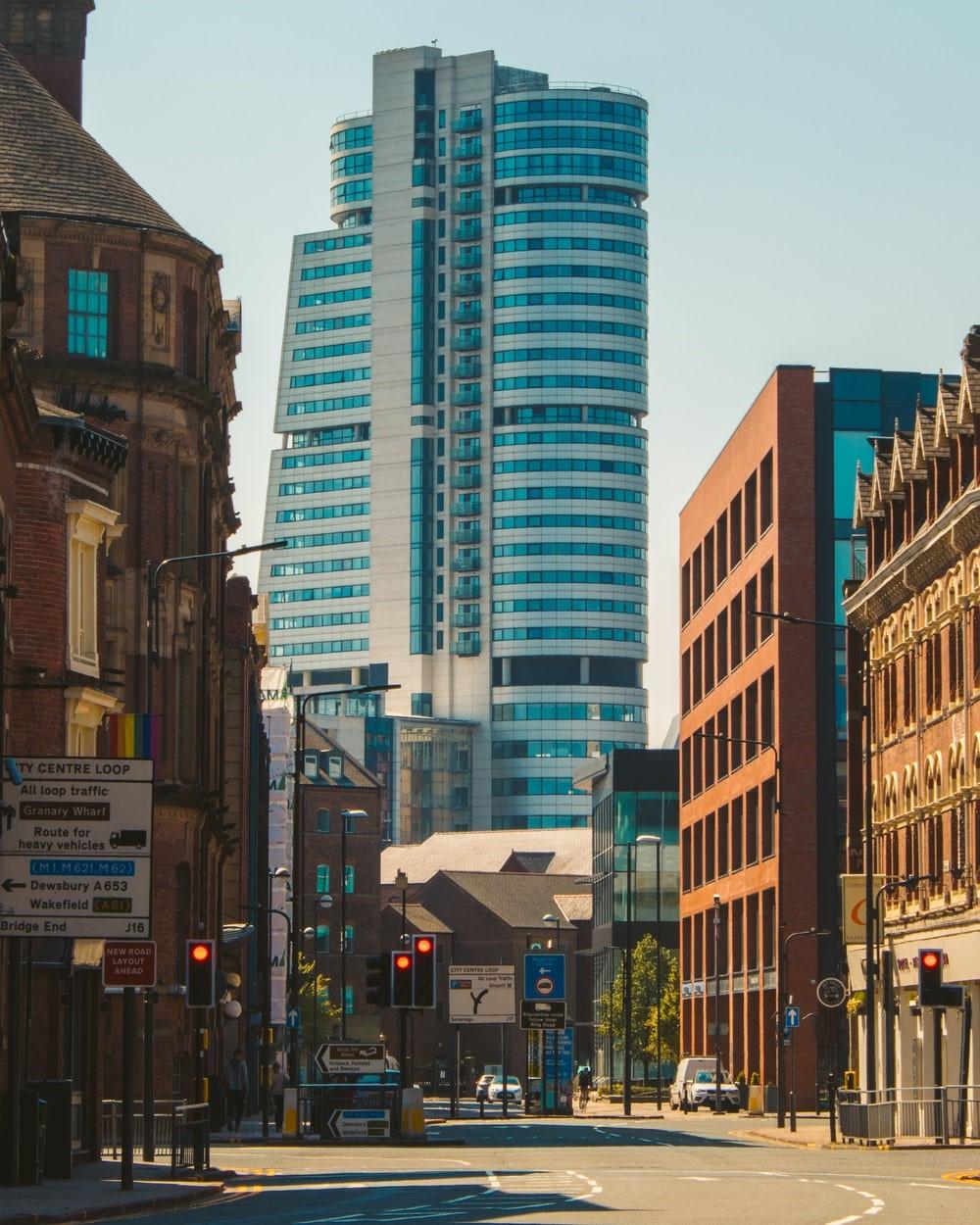


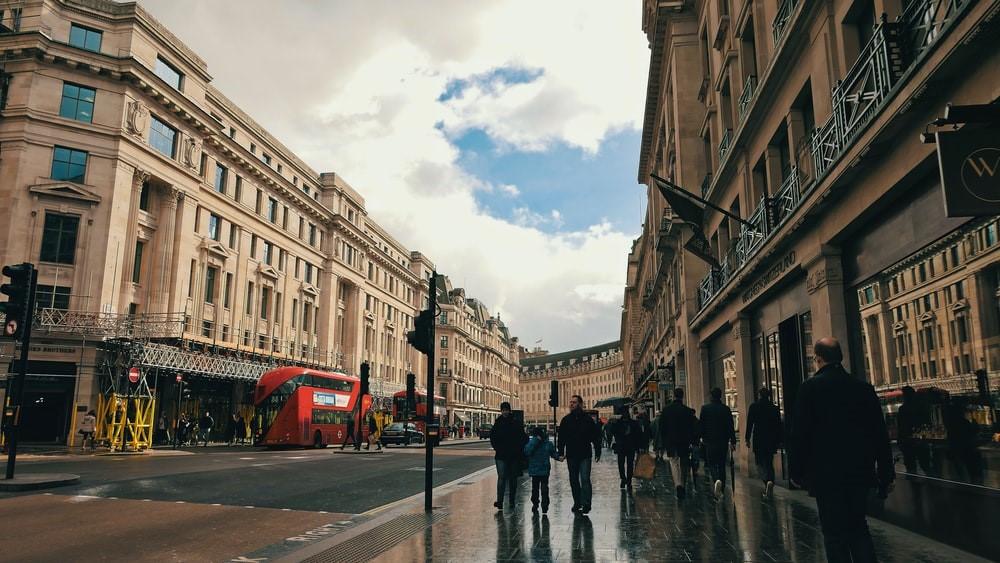


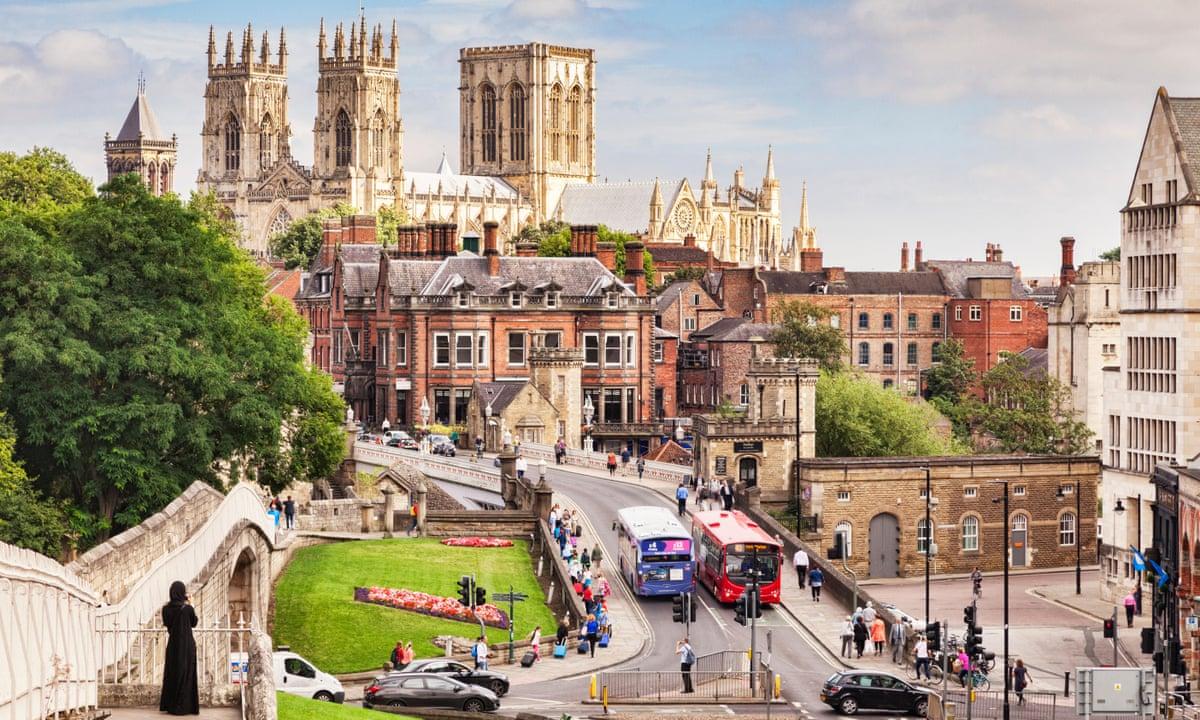


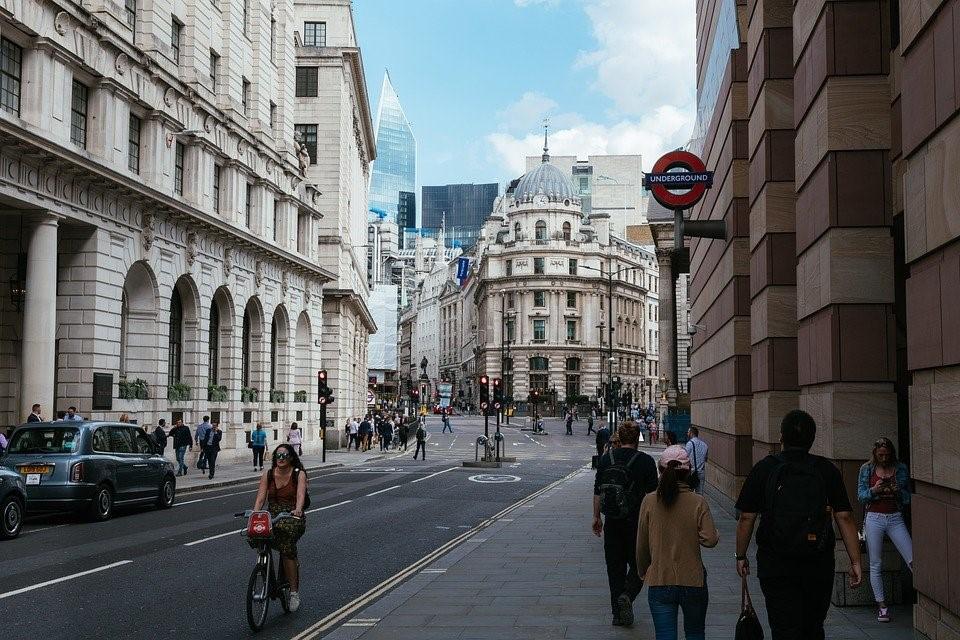


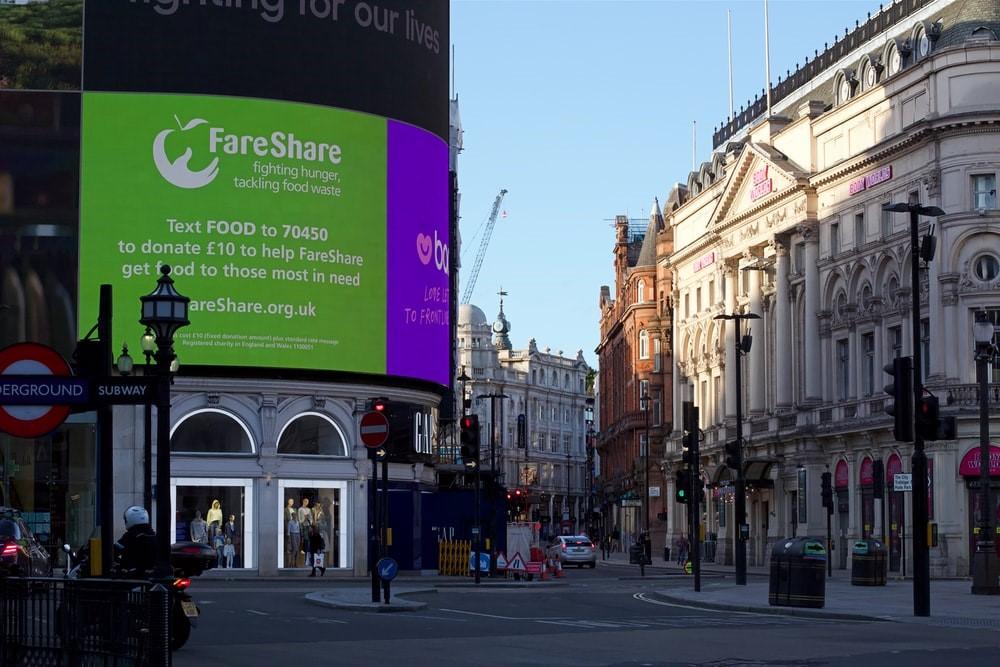


**Supplementary Figure 3.** Scatterplot to show the interaction between experimental condition (1 = Natural condition shown as blue dots, 2 = Urban condition shown as red dots) and dietary restraint (plotted at the 16^th^, 50^th^ and 84^th^ percentile of the restraint distribution) in predicting healthy food desirability ratings (0-100 VAS, higher score indicates greater desirability).
